# “Publish or Perish”: barriers to research publication in an undergraduate medical research program

**DOI:** 10.1186/s13104-023-06542-5

**Published:** 2023-10-13

**Authors:** Abdulrahman F Alsulami, Zeyad O Khaimi, Mohammed A Hadi, Yazeed H Aljabri, Talha S Mayet, Alaa Althubaiti

**Affiliations:** 1 College of Medicine, King Saud bin Abdulaziz University for Health Sciences, P.O. Box 9515, Jeddah, 6656, 21423 Saudi Arabia; 2 King Abdullah International Medical Research Centre, Jeddah, Saudi Arabia

**Keywords:** Medical research, Undergraduate, Students, Publication, Barriers

## Abstract

**Objectives:**

Publication is one of the crucial parameters in research, and the inability to publish has been noted in many medical students’ projects due to different reasons. This cross-sectional study aimed to determine the obstacles that prevented medical students in a health science university from publishing their research from 2018 to 2021. First, an online survey was distributed to assess the obstacles to publication perceived by the medical students. Second, a total of 81 research projects were evaluated by scientific reviewers and their final decision about the publication was recorded.

**Results:**

In total, 162 students filled out the survey. The barriers faced by the students were various. They included an unsupportive research supervisor, a lack of time, an insufficient sample size, and many others. In the reviewer’s evaluation, out of 81 projects, 70 projects (86.4%) were recommended to be published after minor or major modifications, while 11 projects (13.6%) were rejected due to poor writing style, poor results interpretation, and incorrect methodology.

**Conclusion:**

Articulating the barriers to undergraduate medical research publication is important in boosting publication rates and research experience of graduating medical students. Medical research educators and research supervisors should strongly consider creating a framework that tackles existing obstacles and any future matters.

**Supplementary Information:**

The online version contains supplementary material available at 10.1186/s13104-023-06542-5.

## Introduction

Research is essential for the advancement of medicine and the improvement of healthcare. It is also crucial to the practice of evidence-based medicine [1–4]. Medical students play a vital role in the published output and research productivity of any institution [2, 3]. For example, medical students largely contributed to the publications of one German institution, as 28% of the output was authored by students [5]. In addition, exposing medical students to research activities is strongly linked to developing positive attitudes towards research and a tendency to be involved in academic careers later in life. Such exposure is found to be a valuable method to promote health research in the long-term [3]. Even if students do not later pursue a career in research, research experience has been shown to provide students with teamwork skills, knowledge about critical appraisal of literature, and writing skills [2, 3]. Moreover, after writing a paper, nothing motivates students more than getting their work published. Nowadays, fierce competition among graduating medical students creates challenges in securing a position in a decent residency program [3]. One approach used for evaluating doctors is assessing their publishing practices and research skills as not all students can publish their research [2, 6].

### Publication rates

Previous research investigating publication rates and obstacles is limited and shows variable results [2, 6–8]. In one study targeting the medical college of Chennai, 17.4% of the students had their research published [7]. Another British study found that only 14% of students submitted their papers for publication, mainly attributing this small number to a lack of opportunity to conduct a study and a lack of time [2]. Limited research is done locally. A study performed at a local university targeting medical interns resulted in a low rate of student publication, at 3.2%, ascribing it to a lack of support during data analysis, manuscript writing, and financial support [6]. This local percentage is low in comparison with another study conducted in Peru, in which 17.6% of theses were published [8].

Largely, the challenges facing medical students in publishing research remain unexplored, which creates a need for further investigations regarding the rates and obstacles of publication, especially in local institutions. This is likely to increase students’ awareness about the challenges that could decrease their chances of publication.

The medicine program at the College of Medicine at King Saud bin Abdulaziz University for Health Sciences (KSAU-HS) is internationally accredited and aims to enhance the publication trends of undergraduate medical students. As part of the program, students undergo a medical research course for two years where they can select research team members, determine a research topic, and choose a supervisor to provide guidance throughout the course [9]. All of which is facilitated by a panel of research assistants and coordinators. Monitoring publication results is done to enhance the quality of the program. In the campus, the research program has been implemented for four times and has led to a total of 80 publications (i.e., 15% of the total research projects are published).

This study aims primarily at identifying the barriers influencing the publication of research conducted by medical students and presents potential ways to improve outcomes.

## Materials and methods

### Sample, setting, and data collection

The study was conducted in the College of Medicine at KSAU-HS. Secondary and primary data collection methods were utilized. In the secondary data collection approach, the database of students’ research conducted under the medical research course was retrieved from the College. The variables collected were the number of research groups, and the reports of the instructors’ evaluations.

For the survey distribution, a convenience sampling method was used to select participants. All research students that enrolled in the medical research course from 2018 to 2021, (i.e. 4th, 5th, and 6th year medical students) were invited to participate in the study. The survey was developed for this study and can be found in Supplement file 1. Variables in the survey included sex, the topic of the research, and perceived obstacles.

### Data analysis

Descriptive analysis was used to describe the data. Percentages were used to present categorical variables. To determine the challenges faced by medical students in publication, a multiple-response analysis was used. The percent of cases was reported and determined by dividing each count by the total number of cases or respondents choosing an obstacle. Statistical analysis was performed using JMP Pro 14 software (JMP, Pro 14; SAS Institute Inc, Cary, NC, 1989–2019).

## Results

### Participant characteristics

A total of 162 out of 545 students participated in the study reporting the obstacles faced when attempting to publish their research, through the self-administered survey (with a response rate of approximately 30%). Each participant was able to choose multiple answers. The number of male responses was 93 (57.4%), and the number of female responses was 69 (42.6%). The current GPA of the students, which is out of 5, varied with the majority of 133 (82.1%) participants scoring between 4.5 and 5. There were 20 (12.3%) participants whose GPA was between 4 and 4.49. In addition, there were eight (4.9%) participants whose GPA was between 3.5 and 3.99. There was a single (0.6%) participant with a GPA that was less than 2.5. Examples of current research topics include medicine (25.9%), oncology (13.6%), surgery (10.5%), and medical education (4.9%).

Most students used cross-sectional study designs (n = 91, 56.1%), or cohort study designs (n = 55, 34%). In addition, other study designs were also used; these were randomized clinical trials (5.6%), case controls (3.1%), systematic review/meta-analysis (0.6%), and case reports/series (0.6%).

### Obstacles faced in research publication

**Fig. 1:**
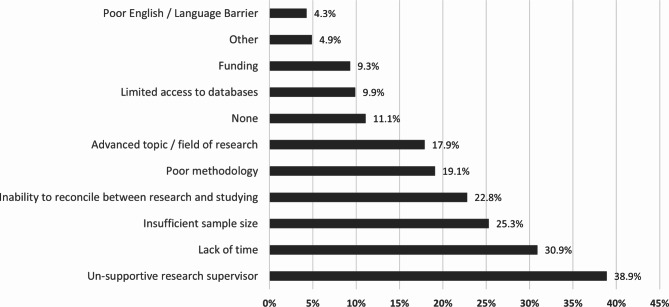
Percentages of responses for obstacles faced in publishing medical research as perceived by students in undergraduate medical research program

Out of 162 students, a total of 18 students reported no obstacles, and 144 students reported at least one obstacle (the total responses to the obstacles question was 315). Figure 1 shows the distribution of obstacles among the 144 students, expressed as the percent of cases. As shown in Fig. 1, a total of 63 participants (38.9%) perceived that an unsupportive research supervisor was a barrier to publishing their research. Lack of time had a total of 50 responses (30.9%) and the inability to reconcile between research and studying had 37 responses (22.8%), representing a large part of the problem that students face. Insufficient sample size was reported by 41 participants (25.3%) and was the third most significant factor.

Poor methodology and choice of an advanced topic or field of research had a small gap between them with 31 responses (19.1%) and 29 responses (17.9%), respectively. Limited access to data, for example, incomplete or unavailable electronic medical records, was reported by 16 students (9.9%), while funding was reported by 15 students (9.3%) and poor English was reported by 7 students (4.3%). Moreover, 4.9% (n = 8) of participants reported other factors. Students were required to elaborate if “other” was chosen, using an open-question format. Their answers were “uncooperative team members” (n = 2), “having difficulties with journals’ websites”, “problems with the results conducted”, “further editing of manuscript”, “bad documentation within old physical record”, and “wanting to publish their work in higher-impact journals”.

### Opinions of reviewers on manuscripts’ publishability

Over the two cohorts that started the medical research program in 2019 and 2020, a total of 93 projects were implemented. All research manuscripts were evaluated by two research reviewers from the College Research Committee. Each research group received feedback from both. To evaluate the research manuscript’s publishability, instructors in the program have added a new evaluation criterion in addition to the graded evaluation of the manuscript. All manuscripts were screened and grouped into three categories of recommendations: recommend publication after minor modification, recommend publication after major modification, and not publishable. This was added to monitor the quality of research and address the reasons. In addition, the program’s instructors considered the final evaluation on publishability to be of the wary reviewer to insure necessary feedback to the students.

**Fig. 2:**
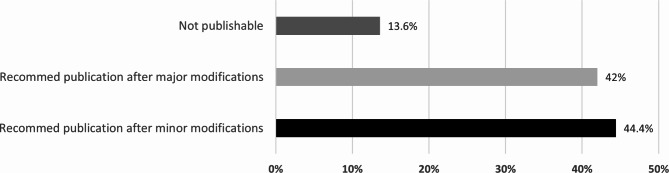
Opinion of reviewers on research manuscripts’ publishability at the end of medical research program (n = 81 research manuscripts)

The summary results of publishability are shown in Fig. 2. Out of the 93 projects, 12 projects had no complete evaluations, were published, or were submitted for publication. They were therefore excluded from the analysis. Results showed that out of the 81 projects, 13.6% (n = 11) of the projects were seen as not publishable by a reviewer, 42% (n = 34) could be published after major modifications, and 44.4% (n = 36) were publishable after minor modifications. For the not publishable projects, the main reasons indicated by reviewers were poor writing styles, poor results interpretation, poor English language (n = 9), and insufficient sample size/incomplete data collection (n = 2), indicating an incorrect methodology. In addition, manuscripts with major modifications needed additional time and effort from the investigators. If this was not accomplished, it was likely that upon submission to the journal, the research manuscript would be rejected and would not be publishable [10]. Addressing the common reasons that prevent manuscripts from publication is important at the early stage of the medical research process.

## Discussion

Identifying the obstacles preventing students from publishing their work is crucial for the educative process and scientific advancement. This study is one of a limited number of research papers discussing and acknowledging these barriers in undergraduate medical curricula and particularly in Saudi Arabian universities [4, 6].

The difference in publication rates over the years, and between the different universities, is attributed to different factors. In our study, the most frequent obstacle was having an unsupportive research supervisor. This could be because most supervisors are full-time physicians and university lecturers. This means that research programs are an added responsibility for them. This seems to be more common than expected in previously mentioned studies. Additionally, a study in Government Medical College, Nagpur, India, amongst 156 medical students, suggested that 68% of these students faced a lack of proper guidance as an obstacle [11].

As anticipated, lack of time and the inability to reconcile research and studying represent a large part of the problem that students face. It was predicted that a significant number of students would report not having enough time to balance medical school and the research publication process. Supporting these findings, a paper conducted by Giri et al. [12] in India showed that 59.5% of the students of the Pravara Institute of Medical Sciences also reported a lack of time due to the curriculum as a barrier in conducting a study. Lack of time was also reported as the main obstacle in a Malaysian study [13].

Insufficient sample size, poor methodology, and choice of an advanced topic or field of research were also important factors. This could be the result of starting the research curriculum immediately after joining the college of medicine, meaning that students may not be knowledgeable enough to start the research process. Poor English was also reported as an obstacle, although the students took 24 h of English courses involving reading, communication skills (i.e. writing and oral skills), and grammar before joining the college.

One of the interesting obstacles reported was problems with results preparation after data gathering. This factor is important because it is one of the fundamental skills of a researcher. Furthermore, KSAU-HS has a set mandatory biostatistics course for all students before they enter the college of medicine. In addition, as part of the research program, students undergo descriptive and inferential statistics classes in addition to practical hands-on sessions. Research conducted in similar settings enrolled 327 students to assess attitudes toward statistics in medical research. These findings suggest that although students can see the benefit of statistics in their professional life, they carry neutral to negative attitudes towards this area [14]. These attitudes may have impacted the willingness of students to develop the results section of their manuscript.

Current recommendations in the literature include providing workshops and courses for research methodology and the publication ethics and process, which is already covered in KSAU-HS as part of the mandatory research curriculum and other activities [4, 10]. Therefore, the obstacles that must be tackled depend on students’ implementation of proper perceived solutions after they have been addressed, such as cultivating time management skills, working with a cooperative supervisor, and improving the required biostatistical skills to plan a proper methodology [6, 14–16].

### Limitations

The study findings presented may be insightful for undergraduate medical students from the two cities Jeddah and Riyadh KSAU-HS campuses, as the same research curriculum is set for both campuses. However, results cannot be generalized to all research programs as every college has its own curriculum and a different approach towards research [17].

For future work, differences in publication rates and obstacles reported by gender, and other factors related to students’ demographics, could be investigated. In addition, due to the COVID-19 pandemic, the teaching of the medical research course has shifted to an online delivery format for three years. It would be interesting to examine the obstacles faced by students in conducting their research and publishing the final manuscript using online learning.

## Conclusion

Overall, medical students reported experiencing numerous obstacles that affect their chances of publishing papers, especially having an unsupportive supervisor, lack of time, and facing issues with sample size. Existing research shows varying and unsatisfying results reflecting the need for deeper exploration. Further research is required to provide a clearer image and solutions that are practical and would aid in overcoming these barriers to improve students’ research conduction and quality.

### Electronic supplementary material

Below is the link to the electronic supplementary material.

Supplementary Material 1

## Data Availability

The datasets used and/or analysed during the current study are available from the corresponding author on reasonable request.

## Abbreviations

KSAU-HS: King Saud bin Abdulaziz University for Health Sciences
