# Supplementary material for: “Publish or Perish”: barriers to research publication in an undergraduate medical research program
Source: BMC Res Notes. 2023 Oct 13;16:269. doi: 10.1186/s13104-023-06542-5 (PMC10571371; doi:10.1186/s13104-023-06542-5)
Supplement: Supplementary file 1 — Supplementary Material 1 [file 13104_2023_6542_MOESM1_ESM.docx]

**Supplement file 1: Questionnaire**

1. You are invited to participate in this study which aims to identify the overall trend of undergraduate student’s publication of research in KSAU-HS, Jeddah.
   - Agree to participate
   - Disagree to participate
2. Sex
   - Male
   - Female
3. From which year are you?
   - 6^th^ year medical student
   - 5^th^ year medical student
   - 4^th^ year medical student
4. What is your current GPA? (Out of 5)
   - 4.5 - 5
   - 4 - 4.49
   - 3.5 - 3.99
   - 3 - 3.49
   - 2.5 - 2.99
   - Less than 2.5
5. What was the research study design?
   - Cross-sectional
   - Case report / series
   - Case control
   - Cohort
   - Randomized Clinical Trial
   - Systematic review / meta-analysis
6. Please mention the research topic/study area:……………………..
7. What are the obstacles that you faced in publishing your research? (Choose as many as applicable)
   - Funding
   - Lack of time
   - Advanced topic / field of research
   - Poor methodology
   - Un-supportive research supervisor
   - Poor English / Language Barrier
   - Limited access to databases
   - Insufficient sample size
   - Inability to reconcile between research and studying
   - None
   - Others (mention them)
